# Identification of a Novel Heterozygous Missense Mutation in the *CACNA1F* Gene in a Chinese Family with Retinitis Pigmentosa by Next Generation Sequencing

**DOI:** 10.1155/2015/907827

**Published:** 2015-05-17

**Authors:** Qi Zhou, Jingliang Cheng, Weichan Yang, Mousumi Tania, Hui Wang, Md. Asaduzzaman Khan, Chengxia Duan, Li Zhu, Rui Chen, Hongbin Lv, Junjiang Fu

**Affiliations:** ^1^Department of Ophthalmology, Affiliated Hospital of Luzhou Medical College, Luzhou, Sichuan 646000, China; ^2^Key Laboratory of Epigenetics and Oncology, The Research Center for Preclinical Medicine, Luzhou Medical College, Luzhou, Sichuan 646000, China; ^3^Department of Molecular and Human Genetics, Baylor College of Medicine, Houston, TX 77030, USA

## Abstract

*Background.* Retinitis pigmentosa (RP) is an inherited retinal degenerative disease, which is clinically and genetically heterogeneous, and the inheritance pattern is complex. In this study, we have intended to study the possible association of certain genes with X-linked RP (XLRP) in a Chinese family. *Methods.* A Chinese family with RP was recruited, and a total of seven individuals were enrolled in this genetic study. Genomic DNA was isolated from peripheral leukocytes, and used for the next generation sequencing (NGS). *Results.* The affected individual presented the clinical signs of XLRP. A heterozygous missense mutation (c.1555C>T, p.R519W) was identified by NGS in exon 13 of the *CACNA1F* gene on X chromosome, and was confirmed by Sanger sequencing. It showed perfect cosegregation with the disease in the family. The mutation at this position in the *CACNA1F* gene of RP was found novel by database searching. *Conclusion.* By using NGS, we have found a novel heterozygous missense mutation (c.1555C>T, p.R519W) in *CACNA1F* gene, which is probably associated with XLRP. The findings might provide new insights into the cause and diagnosis of RP, and have implications for genetic counseling and clinical management in this family.

## 1. Introduction

Retinitis pigmentosa (RP; OMIM 268000) is an inherited retinal degenerative disease that causes the progressive visual loss and often complete blindness [1]. RP is caused by the loss of photoreceptors (rods and cones) and abnormalities in the retinal pigment epithelium (RPE) cells, leading to night blindness, development of tunnel vision, and sometimes loss of central vision [2]. The frequency of RP had been reported as 1 in 3,500 people worldwide [3]. Currently there is no cure for RP and the visual prognosis is very poor. But the progression of the disease can be reduced by proper vitamin A supplementation [4], treating the complications and helping patients to cope with the social and psychological effect of blindness [5]. RP is clinically and genetically heterogeneous, and the inheritance pattern cannot be easily determined because of phenotypic and genetic overlap [6]. RP can be inherited in either an autosomal dominant (ADRP), autosomal recessive (ARRP), X-linked (XLRP), or digenic and mitochondrial mode [2, 3, 7, 8]. Among RP types, XLRP is particularly severe, typically manifested as night blindness with progressive visual loss causing blindness in affected males [9]. The molecular characterization and diagnosis of RP is challenging for many patients due to the high number of genes and variants among other factors involved in RP [10, 11]. Identification of gene-specific phenotypes is essential for the accurate diagnosis and identification of the cause of frequent genetic defects underlying heterogeneous retinal dystrophy [6]. So far, mutations in more than 50 genes have been identified to be associated with RP [11, 12].

Mutations in* CACNA1F* gene have been found to be associated with some retinal disease and are suspected to have link with RP. In this study, we have intended to study the possible association of certain genes with XLRP disease in a Chinese family. By using next generation sequencing (NGS) we have found a novel heterozygous missense mutation in the* CACNA1F *gene probably associated with XLRP.

## 2. Materials and Methods

### 2.1. Clinical Diagnosis and Sample Collection

**Figure 1:**
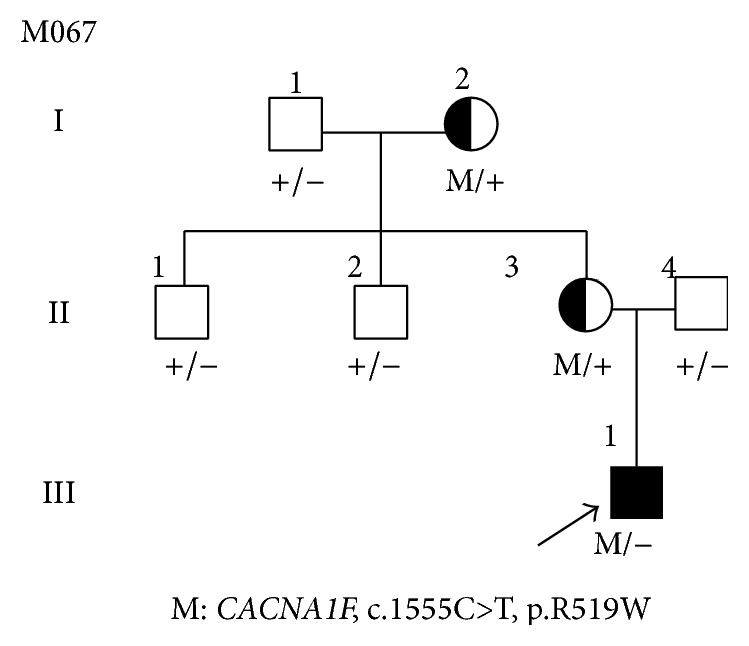
Pedigree M067 structure and segregation of* CACNA1F* mutation in a Chinese RP family. Normal individuals are shown as clear circles (females) and squares (males), affected individuals are shown as filled symbols, and carrier is shown as half-filled circle. The patient above the arrow indicates the proband. “M” indicates mutant allele of* CACNA1F* gene, c.1555C>T, p.R519W, “+” indicates c.C1555 normal allele of* CACNA1F* gene.

A Chinese proband (M067, Figure 1, III: 1) suffering with RP was collected from “Affiliated Hospital of Luzhou Medical College” in Sichuan Province, China. A total of 7 individuals were recruited in this genetic study (Figure 1). All subjects were identified at Luzhou Medical College in Sichuan, China. Full medical and family histories were taken, pedigrees were drawn, and an ophthalmologic examination was performed. Each patient underwent standard ophthalmic examination: best correct visual acuity (BCVA) according to projected Snellen charts, slit-lamp biomicroscopy, dilated indirect ophthalmoscopy, fundus photography, and visual field tests (Carl Zeiss, Germany). Retinal structure was examined by optical coherence tomography (OCT) (Carl Zeiss, Germany). Electroretinograms (ERGs) were performed (RetiPort ERG System; Roland Consult, Wiesbaden, Germany) using corneal “ERGjet” contact lens electrodes. The ERG protocol complied with the standards published by the International Society for Clinical Electrophysiology of Vision. The diagnosis of RP was based on the presence of night blindness, fundus findings (retinal pigmentation, vessel attenuation, and various degrees of retinal atrophy), severe loss of peripheral visual field, abnormal ERG findings (dramatic diminution in amplitudes or complete absence of response), and family history. This study had received approval from the Ethics Committee of the Luzhou Medical College, China. Written informed consents were obtained from all participating individuals or their guardians. Genomic DNA was isolated from peripheral leukocytes using previously described method [13]. As controls, 100 unrelated healthy Chinese individuals were recruited and genomic DNA was isolated.

### 2.2. Design of Capture Panel

A capture panel of retinal disease genes was described previously [7]. This capture reagent was manufactured by Agilent (Agilent Technologies, Santa Clara, CA). The probes covered 4405 exons and corresponding splice junctions of 163 known retinal disease genes, with a total of 1176 Mbp in design region.

### 2.3. Library Preparation and Targeted Sequencing

Illumina paired-end libraries (Illumina, Inc., San Diego, CA) were generated according to the manufacturer's sample preparation protocol for genomic DNA. Briefly, 1 *μ*g of each patient's genomic DNA was sheared into fragments of approximately 300 to 500 bp. The DNA fragments were end-repaired using polynucleotide kinase and Klenow fragment (large protein fragment). The 5′ ends of the DNA fragments were phosphorylated and a single adenine base was added to the 3′ end. Illumina Y shaped index adaptors were ligated to the repaired ends, then the DNA fragments were amplified by PCR for eight cycles, and fragments of 300 to 500 bp were isolated by purification of beads. The precapture libraries were quantified (PicoGreen fluorescence assay kit; Life Technologies, Carlsbad, CA), and their size distributions were determined by a commercial bioanalytical system (Agilent 2100 BioAnalyzer; Agilent Technologies, Santa Clara, CA). For each capture reaction, fifty precapture libraries (60 ng/library) were pooled together. Hybridization and wash kits (Agilent Technologies, Santa Clara, CA) were used for the washing and recovery of captured DNA following the standard manufacturer's protocol. Captured libraries were quantified and sequenced (Illumina HiSeq 2000; Illumina, Inc.) as 100 bp paired-end reads, following the manufacturer's protocols. Illumina sequencing was performed at the BCM-FGI core.

### 2.4. Bioinformatic Analysis of Sequencing Results

Sequence reads were aligned to human genome reference version hg19 by using an aligner (Burrows-Wheeler Aligner, BWA version 0.5.9) [14]. After recalibration and local realignment using the Genome Analysis Toolkit (GATK version 1.0.5974) [15], the refined sequencing results were subjected to variant calling using a toolkit (Atlas2) [16]. Several common variant databases (such as the 1000 Genomes Database (Build 20110521 and 20101123) [17], dbSNP137 [18], NHLBI GO Exome Sequencing Database [19], NIEHS Exome Sequencing Database [20], YanHuang Project Database (http://yh.genomics.org.cn/), and an internal control database of 997 exomes) were used to filter out common polymorphisms with an allele frequency higher than 0.5% in any of the above databases. Variant annotation was performed using ANNOVAR [21] to remove synonymous mutations and RefSeq genes used as reference to coordinate the mutations. SIFT, Polyphen2, LRT, MutationTaster, and MutationAssessor were used to make functional prediction of missense variants [22]. The pathogenicity of novel missense mutations was predicted by dbNSFP [23]. The Human Gene Mutation Database (HGMD) was used to search for known pathogenic mutations.

### 2.5. Mutation Validation and Segregation Tests

The putative mutations detected by NGS were validated by Sanger sequencing. For each identified mutation, DNA sequences were obtained from the UCSC Genome Browser [24]. RepeatMasker was used to mask the repetitive regions [25]. Primer 3 was used to design the primers at least 50 bp upstream and downstream from the mutation [26], and sequences of primers used for the* CACNA1F *gene causative variation were as follows:* CACNA1F*-L: TGACACCCCTTCTGCCCTTTA and* CACNA1F*-R: AGAAGGAATAGGAGGCTGGGG. After PCR amplification, the amplicons (437 bp) were sequenced on an ABI3500 sequencer (Applied Biosystems Inc., Foster City, CA, USA). The DNA materials of other family members were also sequenced by Sanger sequencing to perform segregation test.

## 3. Results 

### 3.1. Clinical Phenotypes

**Figure 2:**
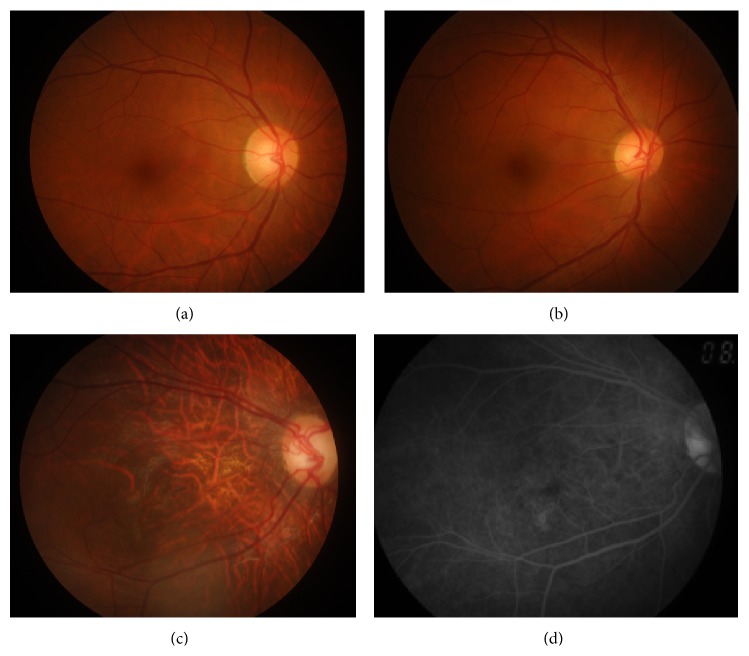
Fundus photograph and fundus autofluorescence of the studied individuals. (a), (b), and (c) indicate fundus photographs in II: 3 (mother), II: 4 (father), and III: 1 (proband), respectively. (d) Fundus autofluorescence in III: 1 (proband), showing tigroid or tessellated features and conus pattern of retina.

**Figure 3:**
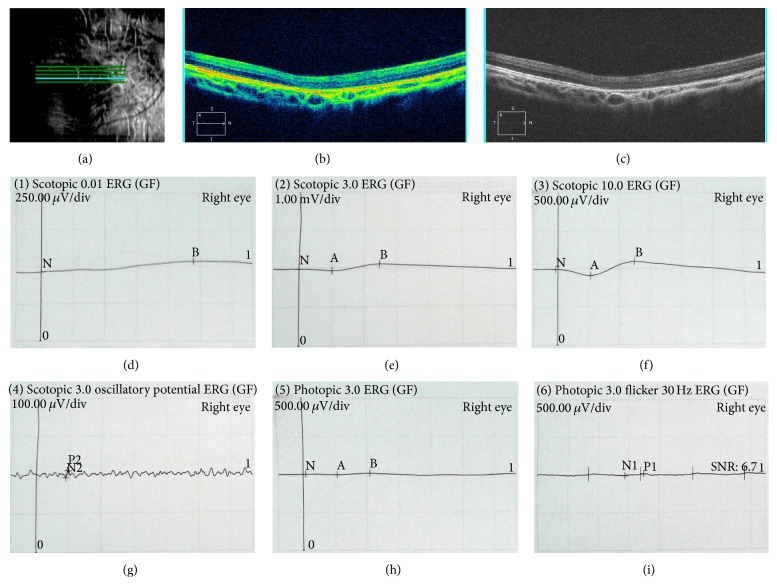
OCT and ERG images of the III: 1. The OCT images showed atrophy of the retina at macula fovea and losing of the fovea ((a), (b), and (c)). Full-field ERG characteristics in right eye ((d), (e), (f), (g), (h), and (i)).

The affected individual (III: 1, Figure 1) presented the early clinical signs of progression in RP at 1+ year old. The proband (III: 1) showed typical fundus features of high myopia, with thinning of the retinal pigment epithelium and the choriocapillaris that resulted in the so-called “tigroid” or “tessellated” appearance of the fundus and pale optic. The fundus features of normal individual (II: 4) and carrier (II: 3) were normal (Figure 2). This observation was further confirmed by OCT imaging of the retina showing foveal atrophy of the retina and losing the normal foveal configuration (Figures 3(a), 3(b), and 3(c)). Electrooculography (EOG) results showed Arden ratio abnormal. ERG results showed A- and B-waves were severely reduced and delayed (Figures 3(d), 3(e), 3(f), 3(g), 3(h), and 3(i)).

### 3.2. Capture Sequencing and Data Processing of Sample

To identify causative mutations in RP patients, we performed targeted capture sequencing of 163 known retinal disease genes using a custom designed capture panel as described in Section 2.2. DNA from affected member (III: 1) was selected, captured, and sequenced. An automatic variant calling, filtering, and annotation pipeline was used to process the capture sequencing data from the sample. We filtered out the common polymorphisms with >0.5% frequency in any of the variant databases queried, including the 1000 Genomes Database (Build 20110521 and 20101123) [17], dbSNP137 [18], NHLBI GO Exome Sequencing Database [19], NIEHS Exome Sequencing Database [20], YanHuang Project Database, and the internal control databases, which were considered too frequent to be pathogenic for RP. Nonpathogenic variations were filtered out by SIFT, Polyphen 2, LRT, MutationTaster, MutationAssessor, and dbNSFP. Sequence variants that were not annotated in any of the above public databases were prioritized for further analysis.

### 3.3. Mutation Screening and Validation

**Figure 4:**
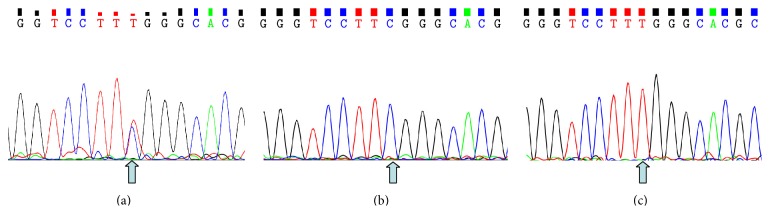
Mutation analysis of* CACNA1F* gene performed by direct sequencing on genomic DNA. (a), (b), and (c) indicate the sequencing results in II: 3 (heterozygous type), II: 4 (wild type), and III: 1 (mutant type), respectively. The arrow indicates the mutation at the nucleotide position c.1555C>T in* CACNA1F* gene.

A heterozygous missense mutation (c.1555C>T, p.R519W) located in exon 13 of the* CACNA1F* gene (GenBank accession number: NM_005183, NP_005174) on X chromosome from the proband was detected, and it was confirmed by Sanger sequencing (Figures 1 and 4(c)), while other known disease-causing gene mutations for RP were excluded. Mutation was not identified in 100 healthy controls. The same heterozygous mutation was subsequently identified in one female carrier (II: 3) of this family (Figure 4(a)), which indicated that the proband (III: 1) was inherited from his mother (II: 3). Further study showed that his grandmother also has the same mutation, revealed by Sanger sequencing (data not shown), suggesting this variant (III: 1) is inherited from his grandmother (I: 2), leading to the pathogenic mutation in offspring male, and showed perfect cosegregation with the disease in the family. The variant was searched in the HGMD and found as a novel mutation, as it was not previously reported. The father of proband (II: 4) and other members of the family are normal with wild type of* CACNA1F* gene (Figures 1 and 4(b) and data not shown).

## 4. Discussion

Genetic sequencing is an important technique that is used to identify genes responsible for a particular phenotype of an organism. It provides important information on genetic function as well as the molecular mechanisms. It can also be used to diagnose and potentially develop treatments for genetic diseases [27]. However, the molecular analysis by using the conventional methods such as Sanger sequencing and arrayed primer extension (APEX) is challenging and cannot be offered routinely. These methods are time consuming and expensive. NGS techniques provide a new approach for a rapid and more efficient way to find disease-causing mutations in affected individuals and to discover new disease genes [7, 28]. In our study, we have applied NGS to find* CACNA1F* gene mutation causing XLRP in a Chinese family.

*CACNA1F* gene (OMIM 300110) is located on chromosome Xp11.23 that consists of 48 exons spanning a genomic region of 28kb.* CACNA1F* gene encodes a multipass transmembrane protein of 1,977 amino acids which is homologous to L-type calcium channel alpha-1 subunits (the Ca_v_1.4 channel) and mediates the influx of calcium ions into the cell [29, 30].* CACNA1F* is expressed in the inner and outer nuclear layers and the ganglion cell layer of the retina [31]. CACNA1F protein contains four homologous domains (I–IV) and each domain is comprised of six transmembrane helical segments (S1–S6) and forms the pore that permits ions to flow down the electrochemical gradient from the extracellular milieu into the cytoplasm [32]. Mutation in* CACNA1F* has been reported to be associated with X-linked congenital stationary night blindness (CSNB), Cone-rod dystrophy-3 (CORDX3), and Aland Island eye disease (AIED) [33–35]. In 20 families with incomplete CSNB, Torben Bech-Hansen et al. [33] identified six different mutations that were all predicted to cause premature protein truncation and indicated that* CACNA1F* mutations trigger a novel mechanism of defective retinal neurotransmission in CSNB patients. In 2 affected members of a French family with the incomplete type of X-linked congenital stationary night blindness (CSNB2), Jacobi et al. [36] identified a 1 bp deletion (C) at nucleotide 4548 in the* CACNA1F*, resulting in a frameshift with a predicted premature termination at codon 1524. Wang et al. [37] found a novel mutation c.[1984_1986delCTC; 3001G>A], p.[L662del; G1001R] in* CACNA1F* in one patient with CSNB. In a large Finnish family with CORDX3, Jalkanen et al. [34] identified a splice site mutation in the* CACNA1F* gene which causes premature termination and deletions of the encoded protein, Ca_v_1.4 alpha-1 subunit. Hauke et al. [38] analyzed a large family of German origin with CORDX and identified a novel large intragenic in-frame deletion encompassing exons 18 to 26 within the* CACNA1F *gene. In affected members with AIED, Jalkanen et al. [35] identified a novel deletion covering exon 30 and portions of flanking introns of the* CACNA1F* gene and this in-frame deletion mutation was predicted to cause a deletion of a transmembrane segment and an altered membrane topology of the encoded alpha-1 subunit of the Ca_v_1.4 calcium channels.

While the mutation in* CACNA1F* gene is mostly associated with the pathogenic alterations of CSNB, CORDX3, and AIED, the phenotype observed in this study is most precisely described as RP-like. Because the OCT and fundus autofluorescence images demonstrated the macular degeneration of patient in this study, which is commonly found in RP patients with CSNB shows qualitatively normal OCT and fundus fluorescein angiography (FFA) images [39]. Furthermore, EOG results showed abnormal Arden ratio; ERG results also showed A- and B-waves were severely reduced and delayed, which are different from CSNB, CORDX3, and AIED. Thus this study indicates that yet another phenotype, XLRP, is also caused by a mutation in the* CACNA1F* gene. Here, we have identified a single nucleotide change c.1555C>T in exon 13 of the* CACNA1F* gene leading to the substitution of arginine by tryptophan (p.R519W) in an individual affected with XLRP. Taken together, the same gene mutations leading to different syndromes or diseases with different phenotypes tell us the importance for gene diagnosis, genetic counseling, and clinical management, such as personalized medicine in our medical genetic practice.

The same heterozygous mutation was identified in normal females (I: 2, II: 3) of this family (Figure 4(a)), which indicates that the mutation in proband (III: 1) was inherited from his grandmother (I: 2), and heterozygous females were carriers with X chromosome linked recessive, which is consistent with previous report [30, 33].

*CACNA1F* is important for the functional assembly and/or maintenance and synaptic functions of photoreceptor ribbon synapses. It helps to release neurotransmitters from nerve terminals initiated by calcium influx through presynaptic voltage-dependent calcium channels. It plays a crucial role in the regulation of tonic glutamate release from synaptic terminals of ribbon synapses in retinal photoreceptors and bipolar cells [39]. Mutations in* CACNA1F* cause abnormal electrophysiological response and visual impairments consistent with a retinal neurotransmission defect. Mutation in this gene also causes the developmental failure or loss of photoreceptor ribbon synapses and consequently profound deficits in synaptic transmission from photoreceptor to second-order retinal neurons [40]. Thus mutation (c.1555C>T, p.R519W) in exon 13 of* CACNA1F* may cause functional abnormality of CACNA1F protein, which is possibly associated with RP development.

## 5. Conclusions

In this study, we have identified a novel heterozygous missense mutation in* CACNA1F* gene (c.1555C>T) in a Chinese RP patient. Currently, the clinical diagnosis of RP is based on the presence of constricted visual fields, night blindness, decreased visual acuity, dark pigmentation in the bone spicules, progressive retinal atrophy, attenuated retinal vessels and fine pigmented vitreous cells, and a reduced or absent electroretinogram. Also, the progress of RP is not consistent; some patients exhibit symptoms from infancy while others may not notice symptoms until later in life. Identification of the responsible gene mutation earlier may aid diagnostic feasibility of RP. Also, this can help in therapeutic research on RP in time. Identification of this mutation (c.1555C>T) in* CACNA1F* gene may have significant contribution for the RP diagnosis, genetic counseling, and clinical management, for example, future treatment strategy in this family.
